# Measured Regional Division Optimization for Acoustic Tomography Velocity Field Reconstruction in a Circular Area

**DOI:** 10.3390/s24062008

**Published:** 2024-03-21

**Authors:** Yixiao Chen, Xinzhi Zhou, Jialiang Zhu, Chenlong Dong, Tao Xu, Hailin Wang

**Affiliations:** 1 College of Electronics and Information Engineering, Sichuan University, Chengdu 610065, China; 15883908629@163.com (Y.C.); npiczhujialiang@126.com (J.Z.); dongcl_0219@163.com (C.D.); 2 Key Laboratory of Nuclear Reactor System Design Technology, Nuclear Power Institute of China, Chengdu 610213, China; xutaonpic@163.com (T.X.); hailin_lie@163.com (H.W.)

**Keywords:** adaptive optimization, acoustic tomography, multipath effect, multiple sub-objectives, velocity field reconstruction

## Abstract

The acoustic tomography (AT) velocity field reconstruction technique has become a research hotspot in recent years due to its noninvasive nature, high accuracy, and real-time measurement advantages. However, most of the existing studies are limited to the reconstruction of the velocity field in a rectangular area, and there are very few studies on a circular area, mainly because the layout of acoustic transducers, selection of acoustic paths, and division of measured regions are more difficult in a circular area than in a rectangular area. Therefore, based on AT and using the reconstruction algorithm of the Markov function and singular value decomposition (MK-SVD), this paper proposes a measured regional division optimization algorithm for velocity field reconstruction in a circular area. First, an acoustic path distribution based on the multipath effect is designed to solve the problem of the limited emission angle of the acoustic transducer. On this basis, this paper proposes an adaptive optimization algorithm for measurement area division based on multiple sub-objectives. The steps are as follows: first, two optimization objectives, the condition number of coefficient matrix and the uniformity of acoustic path distribution, were designed. Then, the weights of each sub-objective are calculated using the coefficient of variation (CV). Finally, the measured regional division is optimized based on particle swarm optimization (PSO). The reconstruction effect of the algorithm and the anti-interference ability are verified through the reconstruction experiments of the model velocity field and the simulated velocity field.

## 1. Introduction

Flow phenomena are widely present in important fields such as industry, agriculture, medicine, national defense, and security, e.g., coolant flow rate monitoring in nuclear power plants, aerodynamic field measurements in combustion chambers, petroleum transportation, natural gas transportation, civil water metering, and so on. Therefore, scientific research on velocity measurement is increasing gradually [1,2].

Traditional flow velocity measurement techniques are often based on contact single-point measurement of flow meters, which tend to produce large deviations in the measurement results compared to the original velocity field, thus affecting the reliability and accuracy of industrial automation systems [3].

Based on the drawbacks of single-point flow velocity measurement techniques, multipoint flow velocity measurement techniques have been further developed, and common flow measurement techniques include particle image velocimetry (PIV), electromagnetic measurement methods, and acoustic tomography (AT). The PIV velocity field reconstruction method uses the trajectories of the particles to represent the laws of the fluid in the flow field. However, releasing tracer particles into the flow field makes it difficult to avoid their interference with the original flow field, and the high cost of the hardware makes the implementation of the experiment difficult [4,5].

The essence of the electromagnetic measurement method is that the fluid moves in the magnetic field, which will generate an induced electromotive force (EMF), and the flow rate information is obtained according to the magnitude of EMF. However, the fluid is required to have strong conductive properties, and there are strong limitations in the scope of application, as it is difficult to apply in distilled water, ethanol, oil fluids, and other non-conductive media [6].

The velocity field reconstruction method based on AT is a typical non-contact method, which utilizes the correspondence between the acoustic wave propagation velocity and the flow velocity to realize the flow velocity measurement, with the advantages of real-time performance, high accuracy, wide range, and strong adaptability [7]. It features neither tracer particles that interfere with the flow field nor dependence on the conductive properties of the flow field, and compared with other measuring instruments, the acoustic transducer is small and easy to install, so it is widely used in more situations, such as narrow industrial pipelines or small boilers [8,9].

However, AT has been applied more to temperature field reconstruction and has not been widely used in velocity fields. Jovanovic [10] introduced the angles of departure/arrival of sound waves into the reconstruction algorithm to reconstruct the wind velocity field. Fan [11] utilized the acoustic wave method to reconstruct a two-dimensional velocity field inside a boiler. However, it is only validated for two flow field models with simpler distributions, so the algorithm’s adaptability to flow fields of higher complexity needs to be further demonstrated. Li [12] designed a wavelet model to reconstruct the boiler velocity field and proposed a regularization scheme to cope with the scarcity of acoustic measurement data. Cui [13] used the least squares method to reconstruct the flow field in the flue of a power station boiler in three dimensions. Manuela Barth [14] utilized a reconstruction algorithm for line-integrated measurements to reconstruct a three-dimensional velocity field by using 16 pairs of acoustic transducers. V.A. Burov [15] et al. used acoustic tomography to reconstruct a two-dimensional velocity field in an ocean with an inhomogeneous velocity distribution, taking into account the time delay of the acoustic signal and the phenomena of acoustic path bending. Yu and Cai [16] simultaneously reconstructed both the temperature and velocity fields using the simulated annealing algorithm. Zhang [17] proposed a radial basis function method incorporating improved Tikhonov regularization for velocity field reconstruction by arranging 16 acoustic transducers. However, the feasibility of simultaneous operation of multiple transducers in a limited space needs to be further verified. Meanwhile, Zhang [18] simultaneously reconstructed the temperature field and velocity field based on nonlinear acoustic tomography (NAT) by utilizing the covariance matrix adaptive evolution strategy (CMA-ES) algorithm considering the acoustic refraction effect, confirming the feasibility and validity of the CMA-ES algorithm in reconstructing the velocity field and temperature field at the same time.

After analysis, it can be seen that the topology of the acoustic transducer can affect the difficulty of solving the inversion problem in acoustic tomography. Based on this, Zhang [19] also optimized the topology of the acoustic transducer array, and he proposed a transducer array optimization algorithm based on the covariance matrix adaptation evolution strategy (CMA-ES) with the linear independence degree (LID) as the optimization objective and 16 acoustic transducer positions as the optimization variables, and the experiments show that this algorithm can improve reconstruction accuracy and stability.

Meanwhile, Hong [20] combined laser absorption spectroscopy with the algebraic reconstruction technique (ART) to reconstruct two-dimensional temperature and concentration fields. Zhang [21] optimized the layout of the damper based on particle swarm optimization (PSO) to improve the damping effect. Zhang [22] optimized the transducer position using the beetle antennae search algorithm (BAS), and the average relative error of the reconstruction result of the uniform temperature field was used as an objective function along with the total number of grids that the sound line passed through.

Existing studies on acoustic velocity field reconstruction tend to be restricted to rectangular areas, but application scenarios for velocity field measurements are often circular areas such as pipelines. Zhou [23] applied various types of multichannel ultrasonic flowmeters to single-bend pipe flow measurement by simulation, but did not reconstruct the flow field. Currently, there are few velocity field reconstructions for circular areas. Wang [24] reconstructed the three-dimensional velocity field of a pipeline based on AT by using simultaneous algebraic reconstruction technique (SART). However, its reconstruction performance depends on the selection of initial values and relaxation factors, so the reconstruction accuracy is not satisfactory and requires a large number of iteration steps, resulting in inefficient reconstruction [25].

Therefore, it is of great significance to design an acoustic velocity field reconstruction algorithm for circular areas. In view of the existing problems, this paper uses AT, based on the reconstruction algorithm of the Markov function and singular value decomposition (MK-SVD) velocity field reconstruction algorithm, and uses a limited number of acoustic transducers to reconstruct the velocity field in a circular area with a radius of 5 m. In this paper, we perform multiple sub-objective adaptive optimization of measured regional division by using the multipath effect under the consideration of the limitation of acoustic transducer emission angle. The whole set of algorithms is validated in four typical model velocity fields and simulated velocity fields.

## 2. At Measurement Methodology

### 2.1. Principle of Acoustic Flow Measurement

The basic principle of acoustic flow measurement is that the acoustic velocity varies with the medium flow velocity in the velocity field, so the flow velocity distribution can be obtained by analyzing the acoustic velocity distribution. On the acoustic path ${TR}_{j}{TR}_{i}$, the time-of-flight (TOF) from the transducer ${TR}_{j}$ to ${TR}_{i}$ is as follows:

(1) $$t_{{TR}_{j}{TR}_{i}}=\int_{L_{{TR}_{j}{TR}_{i}}}\frac{dL}{c+({\vec{v}}_{x}+{\vec{v}}_{y})\cdot {\vec{n}}_{{TR}_{j}{TR}_{i}}}$$

where c is the speed of sound, ${\vec{n}}_{{TR}_{j}{TR}_{i}}\;$ is the unit vector along the acoustic path in the direction of ${TR}_{j}{TR}_{i}$, ${\vec{v}}_{x},{\vec{v}}_{y}$ are the flow velocity components along the *x*-axis and *y*-axis, and $L_{{TR}_{j}{TR}_{i}}$ is the acoustic path length between the transducer ${TR}_{j}$ to ${TR}_{i}$. Similarly, the TOF from the transducer ${TR}_{j}$ to ${TR}_{i}$ can be obtained.

Considering that the speed of sound c is much larger than the medium flow velocity, the time difference between the upstream and downstream flight of ultrasonic along the acoustic path ${TR}_{j}{TR}_{i}$ is as follows:

(2) $$\Delta t_{k}=t_{{TR}_{j}{TR}_{i}}-t_{{TR}_{i}{TR}_{j}}=\frac{-2}{c^{2}}\int_{L_{k}}\left(v_{x}(x,y)\cos\alpha +v_{y}(x,y)\cos\beta \right)dL_{k}\;=\frac{-2}{c^{2}}\int_{L_{k}}v_{x}(x,y)d{L_{k}}^{\prime }+\frac{-2}{c^{2}}\int_{L_{k}}v_{y}(x,y)d{L_{k}}^{\prime \prime }$$

where cos⁡α and cos⁡β  are the cosines of the angle between the horizontal flow velocity component ${\;v}_{x}(x,y)$ and the vertical flow velocity component $\;v_{y}\left(x,y\right)$ of the medium in the acoustic path and the unit vector of the acoustic path, respectively. $d{L_{k}}^{\prime }$ and $d{L_{k}}^{\prime \prime }$ denote the integration segments for further calculation, $d\left[\frac{L_{k}\cos\alpha }{c^{2}}\right]$ and $d\left[\frac{L_{k}\cos\beta }{c^{2}}\right]$, respectively.

**Figure 1:**
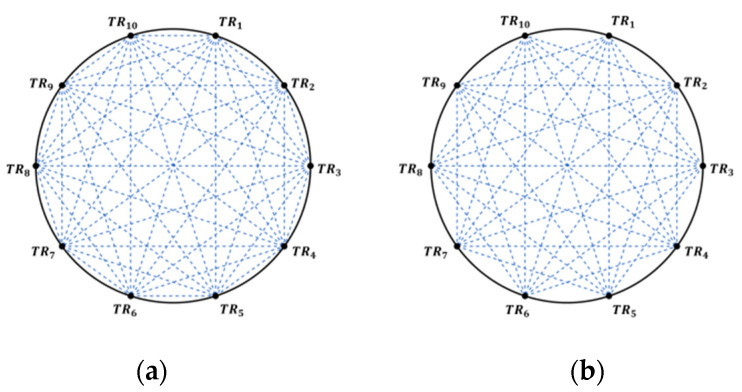
Schematic of velocity field reconstruction in circular area. (**a**) Theoretical reconstruction schematic; (**b**) actual reconstruction schematic.

However, the velocity field tends to be nonuniform, so the multiple paths reconstruction scheme in the temperature field is introduced into the velocity field, and Figure 1a shows the schematic of multipath theoretical reconstruction of the velocity field in a circular area. Multiple acoustic transducers (here ${TR}_{1}-{TR}_{10}$) are arranged uniformly around the area of the velocity field to be reconstructed, thus forming multiple acoustic paths through the area to be measured. Theoretically, when one transducer emits a signal, all other transducers are able to receive the corresponding acoustic signal, producing a total of 45 acoustic paths. However, because acoustic transducers have highly directional emission characteristics, with emission angles typically ranging from $\pm 60^{{}^{\circ}}$, not all of the ideal paths are usable in practice. Figure 1b shows the actual reconstruction schematic after taking into account the emission angle of the acoustic transducers, with 10 transducers generating a total of 35 usable paths.

### 2.2. Principles of Velocity Field Reconstruction Based on MK-SVD

Radial Basis Function (RBF) has the advantages of small computation and high fitting accuracy, and its linear combination can approximate almost any function [26].

Combining the velocity field reconstruction accuracy and computational efficiency, the Markov RBF will be chosen in this paper.

Markov RBF is a radial basis function based on a Markov chain. A Markov chain is a mathematical model that describes a stochastic process of transferring a system between a series of discrete states. It has the Markovian property that future states depend only on the current state and are independent of past states. Therefore, Markov RBF are introduced and applied to a Markov chain to describe the transfer probabilities between states. In the feature space, each data point can be regarded as a state, and the distance between different data points can represent the transfer probability between states.

The Markov RBF as shown in (3) [27]:

(3) $$\varphi_{i}(x,y)=e^{-\varepsilon (\left\|\left(x,y\right)-(x_{i},y_{i})\right\|)}$$

where $\;\left(x_{i},y_{i}\right)$ is the center of the RBF, which, in this paper, denotes the center coordinates of each measured region, ε is the shape parameter of this RBF, and $\left\|\cdot \right\|$ is the Euclidean norm. Thus (2) can be rewritten as follows:

(4) $$\Delta t_{k}=\sum_{i=1}^{N}\omega_{i}\int_{L_{k}}\varphi_{i}\left(x,y\right)d{L_{k}}^{\prime }+\sum_{i=N+1}^{2N}\omega_{i}\int_{L_{k}}\varphi_{i}(x,y)d{L_{k}}^{\prime \prime }$$

where $\omega_{i}$ is the coefficient to be determined. In conjunction with the above, the following is defined:

(5) $$A_{x}=\left(a_{xki}\right)=\int_{L_{k}}\varphi_{i}(x,y)d{L_{k}}^{\prime },\;\;k=1,2,\cdots ,M,\;\;i=1,2,\cdots ,N\;A_{y}=\left(a_{yki}\right)=\int_{L_{k}}\varphi_{i}\left(x,y\right)d{L_{k}}^{\prime \prime },\;\;k=1,2,\cdots ,M,\;\;i=N+1,N+2,\cdots ,2N\;A=\left(a_{ki}\right)=(A_{x},A_{y})\;\omega ={\left(\omega_{1},\omega_{2},\cdots ,\omega_{2N}\right)}^{T}\;t={\left(\Delta t_{1},\Delta t_{2},\cdots ,\Delta t_{M}\right)}^{T}$$

where M denotes the number of effective acoustic paths in the area to be measured and N denotes the number of measured regions. The coefficient matrix A is obtained by calculating the radial basis functions of all acoustic paths, and the matrix t is obtained by measuring and calculating the difference between the upstream and downstream flight times of all acoustic paths, so that (4) can be rewritten in (6):

(6) $$t^{\left(M\times 1\right)}=A^{\left(M\times 2N\right)}\omega^{\left(2N\times 1\right)}$$

From (6), we can see that the solution process of this model is an inverse problem, and SVD is an effective means to deal with matrix inverse problems [28].

The generalized inverse matrix $A^{+}$ of the matrix A is obtained by SVD, and thus the matrix of coefficients to be determined ω in (6) can be expressed as follows:

(7) $$\omega =A^{+}t$$

Substituting the obtained matrix ω into (8), the flow velocity distribution in the area to be measured is obtained:

(8) $$v_{x}\left(x,y\right)=\sum_{i=1}^{N}\omega_{i}\varphi_{i}\left(x,y\right)\;v_{y}\left(x,y\right)=\sum_{i=N+1}^{2N}\omega_{i}\varphi_{i}\left(x,y\right)\;v(x,y)=\sqrt{{v_{x}(x,y)}^{2}+v_{y}{\left(x,y\right)}^{2}}$$

### 2.3. Measured Region Layout for Velocity Field Reconstruction in Circular Area

The way of measured region layout directly affects the matrix A in (5), which ultimately affects the reconstruction effect [29].

At present, most of the research experiments on reconstruction of velocity fields are limited to rectangular areas, and there are few experiments for circular areas, the fundamental reason being that the design difficulty of the measured region layout is much larger compared to the rectangular area: in measured region layout experiments for rectangular areas, the measured region layout is usually divided into m×m grids, (m is a positive integer).

**Figure 2:**
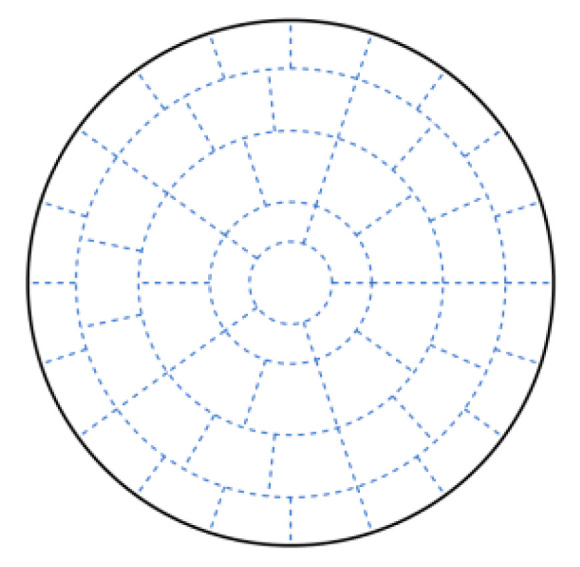
Circular area measured region layout method.

In this paper, we use the Concentric Circle Layout to divide the circular area of velocity field into several measured regions as follows:

Step 1: Divide the radius of the circular area to be measured into n equal parts and draw circles with the center of the circle as the radius, respectively $\frac{1}{n}$, $\frac{2}{n}\cdots \frac{n}{n}$ as the radius;

Step 2: Divide the n−1 concentric circles into $m,2m\cdots m\left(n-1\right)$ equal parts along the radius in the order of smallest to largest radius;

In the above division manner, the circular area is divided into $\frac{mn\left(n-1\right)}{2}$ annular sectors and one circle with the center of the circular area as the center and a radius of $\frac{1}{n}$. The specific division is shown in Figure 2.

## 3. Measured Regional Division Optimization Method for Velocity Field Reconstruction in Circular Area

### 3.1. Acoustic Path Distribution of Velocity Field in Circular Area Based on Multipath Effects

In the acoustic flow velocity measurement technique for circular areas, having more acoustic paths through the area allows more flow velocity information to be captured in the area, thereby enhancing the accuracy of the velocity field reconstruction. However, increasing the number of acoustic transducers, although it can increase the number of acoustic paths to some extent, will enhance the cost.

The multipath effect is the creation of multiple paths in the propagation of a signal, resulting in multiple different propagation times and phases when the signal reaches the receiver. A signal propagated through multiple paths can increase the signal strength received at the receiving end, improving the coverage and reliability of the signal.

In order to utilize more acoustic paths with a limited number of acoustic transducers, in this paper, on the basis of the direct acoustic waves generated between the acoustic transducers, we utilize the once-reflected acoustic waves generated by the multipath effect to improve the utilization of the path information and to further improve the accuracy of the velocity field reconstruction [30].

**Figure 3:**
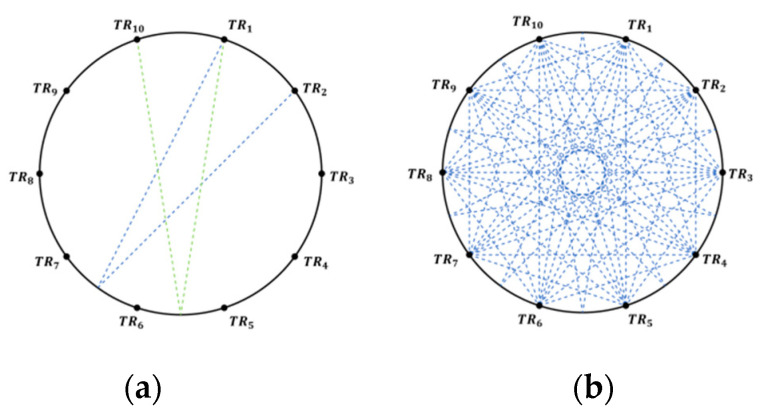
Acoustic paths based on multipath effect. (**a**) Reflected acoustic paths of a transducer; (**b**) overall acoustic paths based on multipath effect.

Figure 3a represents the path distribution of an acoustic transducer within the emission angle, where the emitted acoustic wave is reflected by the opposite region boundary and finally received by the neighboring acoustic transducers.

The 10 acoustic transducers form a total of 10 effective acoustic paths based on the multipath effect, which, when added to the actual direct acoustic paths generated, produce a total of 45 measurable acoustic paths through the area to be measured. Figure 3b represents all acoustic paths based on multipath effects [31].

### 3.2. Multiple Sub-Objectives Optimization for Measured Regional Division

In the process of acoustic reconstruction of the velocity field, the topology of the acoustic transducers and the layout of the measured region have a great influence on the reconstruction effect of the velocity field: the topology of the acoustic transducers directly determines the distribution of the effective acoustic paths as well as the effectiveness of the information acquisition of the velocity field, and the layout of the measured regions directly determines whether the distribution of the acoustic paths is average or not in each measured region, and also influences the sparsity of the coefficient matrices [12].

However, of the existing experiments on the optimization of acoustic velocity field reconstruction, most are optimized for the acoustic transducer topology in the square region, which has a large number of drawbacks, even though it can achieve a higher accuracy of the reconstruction.

First of all, in the existing practical industrial environment, the transducer has usually been uniformly arranged around the area to be measured, and if the optimized transducer topology is applied in engineering practice, it will be modified from the physical structure of the industrial equipment, which will greatly increase the industrial cost. Second, if optimization is performed without considering the fact that transducers have highly directional emission characteristics, even if a topology more conducive to the reconstruction of the velocity field can be obtained theoretically, it is often impossible to apply it in practical industrial applications. Moreover, due to the large number of optimization variables, the optimization results can easily fall into the local optimum, and the optimization efficiency is not high, which ultimately fails to realize the effective improvement of reconstruction accuracy.

Therefore, to address the above shortcomings regarding the optimization of the transducer topology, this paper proposes a multiple sub-objectives optimization algorithm for measured regional division of acoustic velocity field reconstruction in circular areas.

#### 3.2.1. Design of Optimization Variables

**Figure 4:**
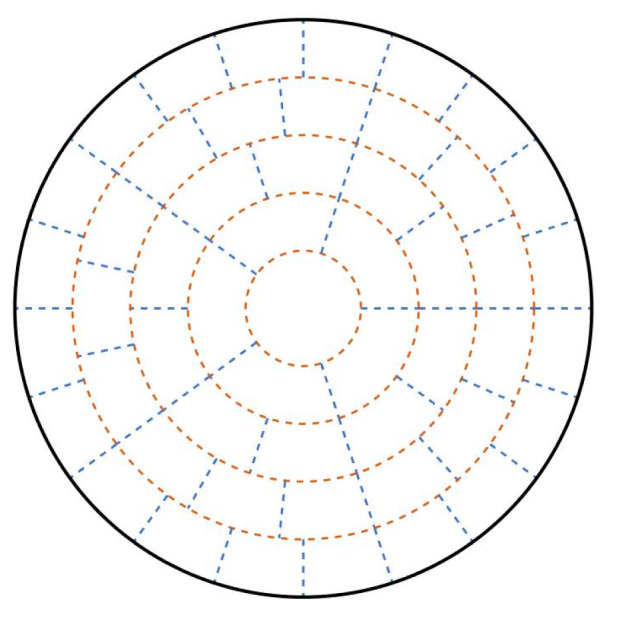
Schematic of optimization variables.

In terms of optimization variables, in this paper, we will optimize the radii of the four concentric circles (orange circles) in Figure 4 on the basis of the Concentric Circle Layout described in the previous section.

Compared with the optimization of the transducer topology, the number of this optimization variable is only four, and it is not easy to fall into the local optimum during the optimization process. Secondly, the impact of the change of the optimization variables is only reflected in the algorithm itself, which can improve the reconstruction accuracy without changing the original industrial topology arrangement. Therefore, the optimization algorithm for measured region division is more achievable.

#### 3.2.2. Design of Optimization Objectives

In terms of optimization objectives, the optimization objectives designed by the method include two sub-objectives, which are the condition number of coefficient matrix A and the uniformity of acoustic path distribution.

- The condition Number of Coefficient Matrix ***A***

In the reconstruction of the flow velocity field, the coefficient matrix A is ill-conditioned, and its degree of ill-health will directly affect the stability of the inversion result. The condition number of matrix is an important indicator to measure whether the matrix is ill-conditioned and the degree of ill-health. For any matrix, the condition number of a matrix A is equal to the product of the 2-norm of A and the 2-norm of $A^{-1}$, it is defined as (9):

(9) $$cond\left(A\right)=\left\|A\right\|\cdot \left\|A^{-1}\right\|$$

The larger the condition number, the more severely ill-conditioned the matrix is, which also means that it is more sensitive to small perturbations in the measurement. In velocity field reconstruction, in order to ensure the stability of the inversion results, the measured region division must be designed so that the condition number of the coefficient matrix is as small as possible.

Therefore, in this paper, the condition number of coefficient matrix is used as the first sub-objective for the measured regional division optimization, and the expression is shown in (10):

(10) $$E_{1}=cond\left(A\right)$$

The smaller $E_{1}$ is, the lower the degree of matrix ill-conditioning in the inversion is and the better the stability of the velocity field reconstruction results.

- The Uniformity of Acoustic Path Distribution

In velocity field reconstruction, inhomogeneous distribution of acoustic paths leads to unstable inversion problems and low reconstruction accuracy, and ultimately an imbalance of reconstruction accuracy in different regions within the velocity field.

Therefore, in this paper, the uniformity of acoustic path distribution is taken as the second sub-objective, which is defined as the value of the variance of the length of the acoustic paths passing through all the measured regions with (11).

(11) $$E_{2}=\frac{\sum_{i=1}^{n}{\left(path\left(i\right)-mean\_path\right)}^{2}}{n-1}$$

where n denotes the number of measured regions, $path\left(i\right)$ is the sum of the lengths of the acoustic paths through the ith region, and mean_path is the average value of the acoustic path lengths of the measured regions under the current division.

The smaller the $E_{2}$ is, the more uniformly the acoustic paths are distributed for each measured region, the more complete the flow velocity information of the circular area is sampled, and the better the reconstruction result is.

A total objective function is generated by linearly weighted summation of the above two sub-objectives [32], as shown in Equation (12):

(12) $$E=\omega_{1}E_{1}+\omega_{2}E_{2}$$

where $\omega_{1}$ and $\omega_{2}$ are the weight sizes of the two sub-objectives in the total objectives, respectively, characterizing the degree of contribution of each sub-objective to the optimization objectives. Optimization of subregion delineation can be achieved by minimizing E, thus improving the accuracy of velocity field reconstruction.

In this paper, CV is used to solve the weight coefficient of each sub-objective [33]. CV is a commonly used multi-criteria decision-making method, which determines the weight of each sub-objective by calculating the coefficient of variation of each sub-objective.

CV is a data-based weight determination method, and its basic idea is to determine the weight of each subgoal by calculating its coefficient of variation. The larger the coefficient of variation, the greater the volatility of the sub-objective, indicating that it carries more information. Therefore, a larger coefficient of variation of a sub-objective will lead to a larger influence of the sub-objective on the overall objective, which ultimately leads to a larger weight.

The specific implementation process is described below:

First, by randomly assigning values to the optimization variables, a certain number of samples for the division of measured region are obtained, and the $E_{1}$ and $E_{2}$ corresponding to each sample are calculated to make a sub-objective dataset. In order to reduce the influence of outliers on the results, and taking into account that the order of magnitude of the sub-objectives may have a large difference, the dataset was standardized with mean 0 and variance 1, and the mean ${mean\_E}_{1}$, ${mean\_E}_{2}$, and the standard deviation ${\;std\_E}_{1}$, ${std\_E}_{2}$ were calculated for the standardization of each index.

Secondly, the coefficient of variation is the ratio of the standard deviation of the indicator to the mean, and the coefficient of variation of the two indicators ${CV}_{1}$, ${CV}_{2}$ are obtained by using (13).

(13) $${CV}_{1}=\frac{{std\_E}_{1}}{{mean\_E}_{1}}\;{CV}_{2}=\frac{{std\_E}_{2}}{{mean\_E}_{2}}$$

Finally, the normalized values of ${CV}_{1}$, ${CV}_{2}$ are taken as the weights of the corresponding sub-objectives, which are expressed as in (14):

(14) $$\omega_{1}=\frac{{CV}_{1}}{{CV}_{1}+{CV}_{2}}\;\omega_{2}=\frac{{CV}_{2}}{{CV}_{1}+{CV}_{2}}$$

- Design of Optimization Process

Based on the above optimization variables and optimization objectives, this paper adopts PSO for automatic optimization search for measured region division [34,35].

In PSO, each solution to the problem can be viewed as a particle, and each particle has two attributes: velocity and position. The particle swarm algorithm assigns initial positions and velocities to all particles, and in each subsequent iteration, the particles update their velocities and positions by tracking the current optimal position pbest of themselves and the optimal position gbest of the entire particle swarm.

For a d-dimensional particle swarm in the kth iteration, the iterative process is represented by (15):

(15) $$\left\{\begin{array}{c} v_{ij}^{k}=\omega v_{ij}^{k-1}+c_{1}r_{1}\left({pbest}_{ij}-x_{id}^{k}\right)+c_{2}r_{2}\left({gbest}_{ij}-x_{id}^{k-1}\right)\\ x_{ij}^{k}=x_{ij}^{k-1}+v_{ij}^{k} \end{array}\right.$$

The $v_{ij}^{k}$ in (15) denotes the velocity of the ith particle in the jth dimension, and $x_{ij}^{k}$ denotes the position of the *i*th particle in the *j*th dimension.

ω denotes the degree of inheritance of the particle to the current velocity, $c_{1}$, $c_{2}$ denote the maximum step of regulation, and $r_{1}$, $r_{2}$ are random numbers between 0 and 1, which increase the randomness of the optimization. A larger ω is good for jumping out of the local optimum, and a smaller ω is good for the convergence of the optimization algorithm.

## 4. Simulation and Analysis

In this paper, the simulation experiment is carried out in a circular area with a radius of 5 m. The distribution of 10 acoustic transducers is uniformly installed around the circumference. The setting of Markov RBF parameters and the way of dividing the entire circular area to be measured have some influence on the efficiency of the optimization algorithm. However, in comparison, the number of measured regions has a greater impact. The performance of the optimization algorithm increases with the number of measured regions, but when the number of regions increases to a certain value, the effectiveness of the optimization algorithm no longer increases. The computational efficiency, however, continues to decrease as the number of measured regions increases. Therefore, synthesizing the two aspects of reconstruction effect and computational efficiency, in this paper, both m and n take the value of 5, and utilizing the Concentric Circle Layout, the entire circular area to be measured is divided into 51 measured regions, with the Markov RBF parameter set to 0.65.

Circular area velocity field reconstruction is usually applied in the context of circular pipe velocity field measurements, whereas the medium inside a circular pipe is usually turbulent, forming vortices or eddies.

Therefore, this paper carries out experimental validation based on four typical vortex velocity fields [36] (single-vortex symmetric field, single-vortex asymmetric field, double-vortex symmetric field, and double-vortex asymmetric field, which are later referred to as velocity fields 1, 2, 3, and 4, respectively).

The expression for the velocity distribution of a single vortex is shown in (16) and the specific parameter settings are shown in Table 1.

(16) $$(x,y)\left\{\begin{array}{c} v_{x}\left(x,y\right)=vexp\times \left\{\frac{-1}{2\sigma^{2}}\times M\right\}cos\beta \\ v_{y}\left(x,y\right)=vexp\times \left\{\frac{-1}{2\sigma^{2}}\times M\right\}sin\beta \end{array}\right.\;\left\{\begin{array}{c} cos\beta =\frac{-(y-y_{0})}{\sqrt{{\left(x-x_{0}\right)}^{2}+{\left(y-y_{0}\right)}^{2}}}\\ sin\beta =\frac{\left(x-x_{0}\right)}{\sqrt{{\left(x-x_{0}\right)}^{2}+{\left(y-y_{0}\right)}^{2}}}\\ M={\left(x-x_{0}\right)}^{2}+{\left(y-y_{0}\right)}^{2}\\ +R^{2}-2R\sqrt{{\left(x-x_{0}\right)}^{2}+{\left(y-y_{0}\right)}^{2}} \end{array}\right.$$

**Figure 5:**
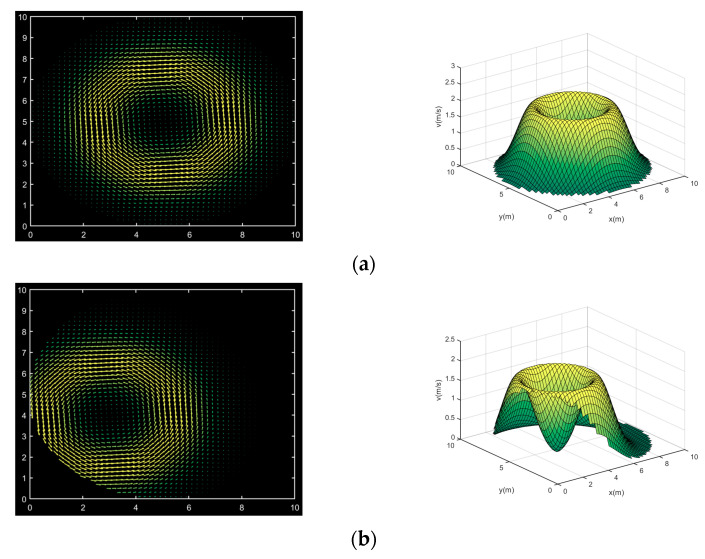

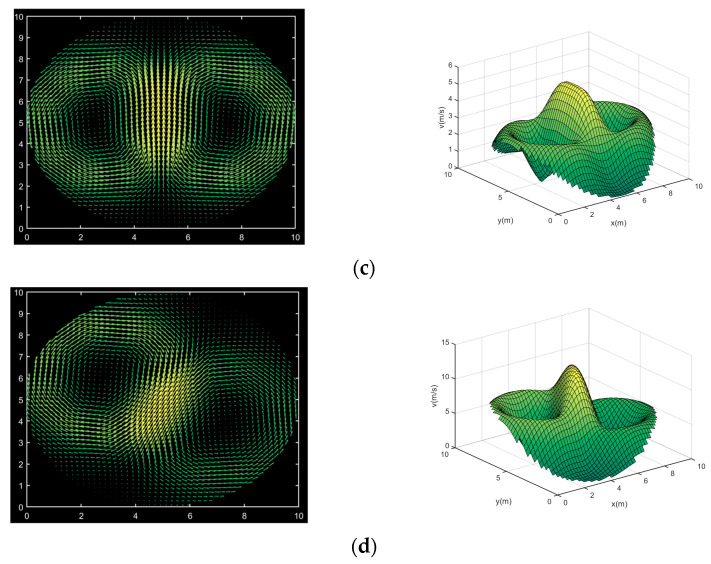
Model velocity fields. (**a**) Model velocity field 1; (**b**) model velocity field 2; (**c**) model velocity field 3; (**d**) model velocity field 4.

In (16), v is the relative amplitude of the solenoidal velocity component, $\left(x_{0},y_{0}\right)$ and R are the center and radius of the vortex, respectively. σ is the standard deviation of the velocity field. β is the angle between the direction of the velocity and the positive direction of the *x*-axis. In order to visualize the above four model velocity fields, their velocity distributions are plotted separately, as shown in Figure 5.

**Figure 6:**
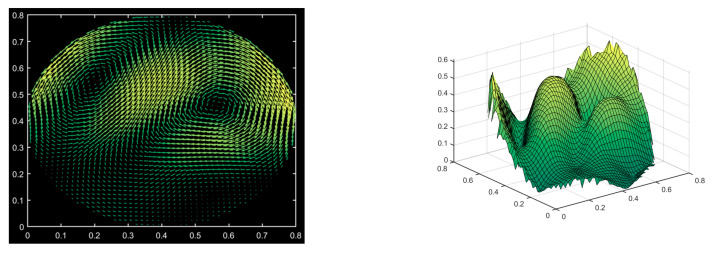
Simulated model velocity field.

At the same time, in order to better prove the effectiveness and versatility of the algorithm, this paper is based on the simulation model of the coolant velocity field in the main pipe of the “Hualong-1” reactor established by the finite element numerical simulation method, as shown in Figure 6, to carry out the validation experiment. The radius of the simulation pipe is 0.4 m.

The reconstruction performance of the experiment was assessed by the root-mean-square error ($E_{rms}$) of the velocity field reconstruction result, which is expressed as (17) [37]:

(17) $$E_{rms}=\frac{\sqrt{\sum_{i}^{n}{\left({VR}_{i}-{VM}_{i}\right)}^{2}}}{\sqrt{\sum_{i}^{n}{{VM}_{i}}^{2}}}$$

where n is the total number of velocity calculation points in the area to be measured, ${VM}_{i}$ is the velocity value of the ith velocity calculation point in the velocity field model, and ${VR}_{i}$ is the velocity value of the ith velocity calculation point in the reconstructed velocity field.

### 4.1. Reconstruction for Velocity Field in Circular Area Based on Multipath Effect

#### 4.1.1. Experimental Validation Based on Model Velocity Fields

In this section, the velocity field reconstruction is realized for each of the four modeled velocity fields shown in (16) with or without considering the multipath effect.

Table 2 demonstrates $E_{rms}$ of the reconstruction for the four velocity fields, with reconstructed image pixels 100 × 100.

Overall, the reconstruction based on the multipath effect is better than the reconstruction considering only the direct acoustic paths. $E_{rms}$ is reduced to different degrees in all four velocity fields, and its four-field average error is 0.7754% lower than that of the reconstruction considering only the direct acoustic paths.

The effect of the reconstruction based on the multipath effect is most pronounced in velocity field 2, where the reconstruction error is reduced by 0.9058%.

Observing the velocity field 2 shown in Figure 5, we can observe that the velocity change is the fastest among the four typical velocity fields, and its large velocity gradient also makes it necessary to have more acoustic paths through the circle area to extract as much velocity information as possible for a more accurate reconstruction. Considering the multipath effect of acoustic paths can improve the utilization of path information, which can effectively improve the above problems.

#### 4.1.2. Experimental Validation Based on Simulated Velocity Field

Table 3 demonstrates $E_{rms}$ of the reconstruction for the simulated velocity field taking into account the multipath effect, with reconstructed image pixels 200 × 200.

The results show that the reconstruction effect based on multipath effect is still better than the reconstruction effect considering only direct acoustic waves in the case of more complex pipeline velocity distribution. The $E_{rms}$ is reduced by 0.5913%, which verifies the validity of the reconstruction of velocity field based on multipath effect and its feasibility in the actual industrial velocity field.

In summary, the velocity field reconstruction based on multipath effect utilizes more acoustic paths with a limited number of transducers, which not only reduces the cost of industrial applications but also makes the acquisition of velocity information more comprehensive, and its reconstruction effect is superior to that of only considering the direct acoustic paths, which realizes a more accurate reconstruction of the velocity field.

### 4.2. Measured Regional Division Optimization of Velocity Field Reconstruction in Circular Area

#### 4.2.1. Experiments Based on Model Velocity Fields

In this section, the number of samples for measured region delineation is 2000, the number of particles of PSO algorithm is 50, and the number of iterations is 50. ω is 0.9 and $c_{1},c_{2}$ are 1.

From the above experimental conditions, the weights of the two sub-objectives in the objective E are calculated as $\omega_{1}$ = 0.3604 and $\omega_{2}$ = 0.6396, which results in (18) for the objective function E:

(18) $$E=0.3604E_{1}+0.\;6396E_{2}$$

Combining the objective function E minimum as the optimization objective and the four optimization variables proposed in Section 3.2.1, PSO is used to optimize the division of the measured region within the circular area, and the radii of the five concentric circles after optimization are thus 0.7849, 1.5408, 2.8968, 4.0837, and 5.

**Figure 7:**
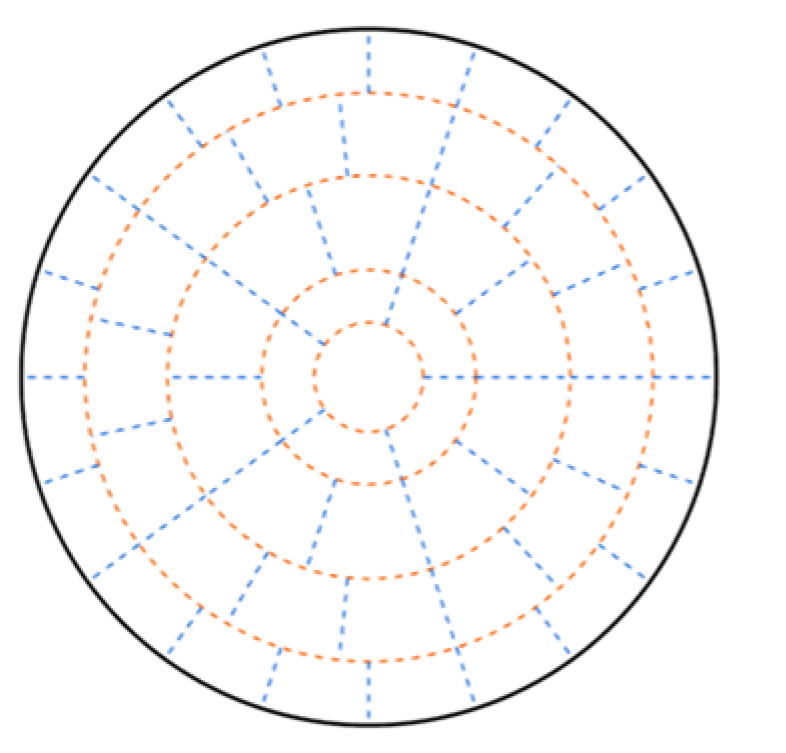
Circular area measured region layout after optimization.

Circular area measured region layout after optimization is shown in Figure 7.

Comparing the results of the measured region layout before and after optimization, it can be seen that the area after optimization decreases in the layers 1, 2, and 5, while the area of layers 3 and 4 increases. Analyzing the reason, it can be seen that before optimization, the distribution of acoustic paths was more concentrated in layers 3 and 4, while the distribution was sparse within layers 1, 2, and 5. After optimization, the distribution of acoustic paths is more uniform.

- Accuracy Verification of the Optimization

**Figure 8:**
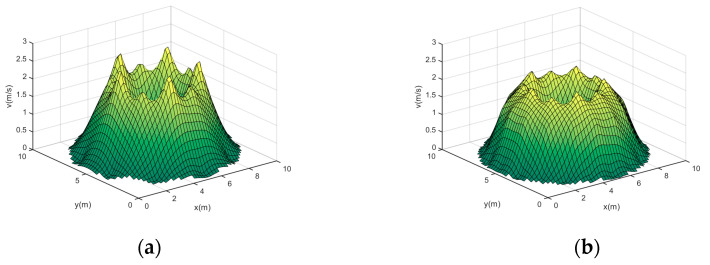

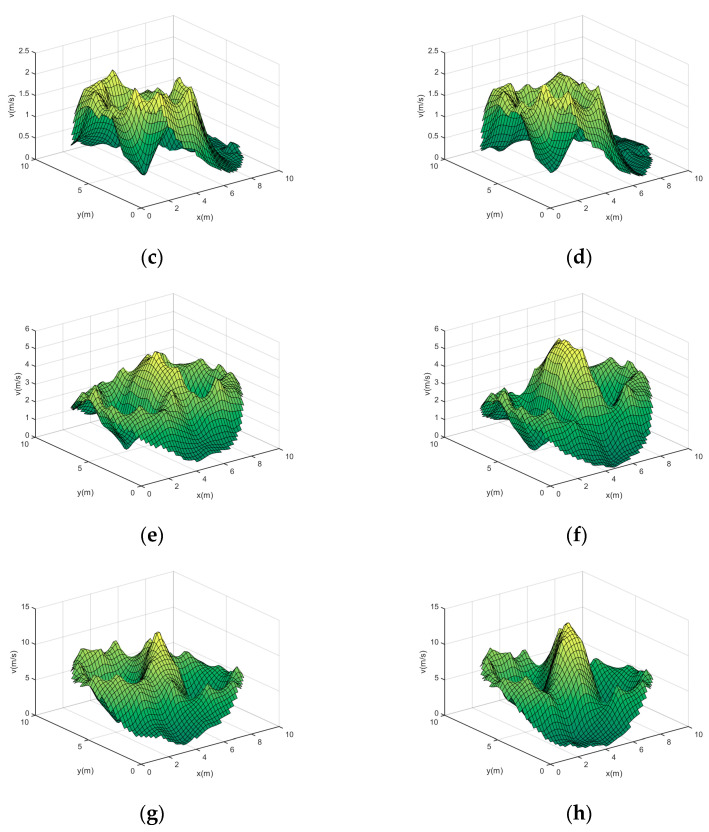
Reconstruction results of model velocity fields before and after optimization. (**a**) Velocity field 1 before optimization; (**b**) velocity field 1 after optimization; (**c**) velocity field 2 before optimization; (**d**) velocity field 2 after optimization. (**e**) velocity field 3 before optimization; (**f**) velocity field 3 after optimization; (**g**) velocity field 4 before optimization; (**h**) velocity field 4 after optimization.

Based on the optimized subregion division results, four velocity fields shown in (16) are used to carry out the accuracy experiments of the optimization method, and the reconstructed velocity distributions before and after optimization are shown in Figure 8.

Analyzing Figure 8, we can see that the optimized reconstruction results can better restore the original velocity fields: for velocity fields 1 and 2, the optimized reconstruction results are smoother in the high velocity region, which is closer to the velocity distribution of the ideal model; for velocity fields 3 and 4, the optimized reconstruction results can restore the distribution of the two vortices more significantly, both for the high velocity and the low velocity distribution region, and its reconstruction results are closer to the ideal model. The main reason is that the optimized acoustic paths are more uniformly distributed for each measured region, so that the contribution of each region is balanced in the process of fitting the velocity fields, thus ensuring the accuracy of the reconstruction.

Table 4 demonstrates $E_{rms}$ of the reconstruction for the four velocity fields before and after optimization, with reconstructed image pixels 100 × 100.

From Table 4, it can be seen that the optimization algorithm is effective within all velocity fields, and the four-field average error is reduced by 2.0740%. Among them, the optimization effect is the best for velocity fields 3 and 4, whose $E_{rms}$ decreased by 2.4938% and 3.5587%, which is mainly due to the fact that the velocity distributions of the two fields mentioned above are more complicated and are more affected by the uniformity of acoustic path distribution, and thus more significant reconstruction effects can be obtained through optimization. It can be concluded that the measured region division obtained using the optimization method proposed in this paper has good results in the reconstruction of circular velocity fields.

- Influence of Noise on Reconstruction Results

The optimized region division should also have good stability in order to ensure high-precision reconstruction of the velocity field in noisy environments [38], and the stability validation experiments will incorporate three different degrees of Gaussian white noise in TOF, with signal-to-noise ratios of 25 dB (low noise), 20 dB (medium noise), and 15 dB (high noise).

$E_{rms}$ of the reconstruction of the four typical velocity fields in (16) before and after the optimization of the measured region division under different noise levels are shown in Table 5, with reconstructed image pixels 100 × 100. In order to make the experimental results more realistic and accurate, all reconstruction results were averaged after 20 calculations.

**Figure 9:**
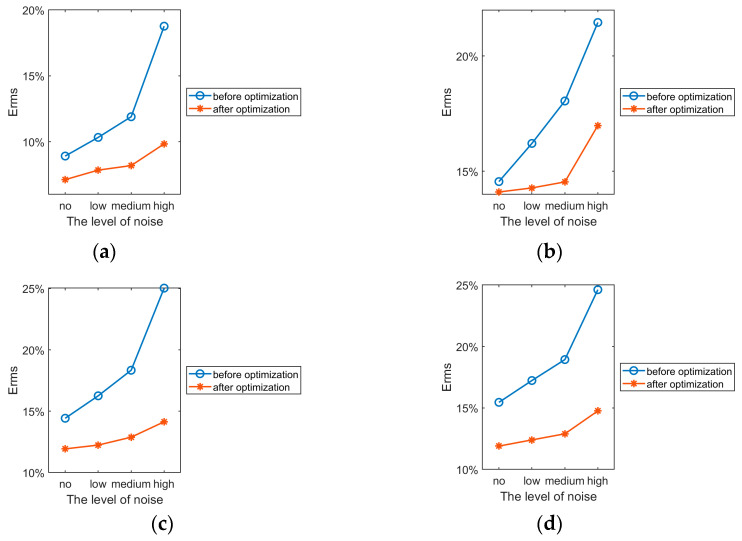
Curve of the mean value of the error at different noise levels. (**a**) Velocity field 1; (**b**) velocity field 2; (**c**) velocity field 3; (**d**) velocity field 4.

The variation curves of the average value of reconstruction error for each field before and after optimization at different noise levels are shown in Figure 9. The flow field reconstruction results before and after optimization at different levels of noise are presented in the Appendix A.

From Table 5 and Figure 9, we can see that for each velocity field, the higher the noise, the higher the reconstruction error. Under different noise levels, the reconstruction of all velocity fields using the optimized measured region division is better than that before optimization, and the stability is also better.

For the low noise level, the average error of the optimized four fields is reduced by 3.3148%, and the reconstruction error for velocity fields 2, 3, and 4 before optimization is already more than 15%, which makes it impossible to realize accurate reconstruction, but the reconstruction effect after optimization is not much affected by the noise, and we can still reconstruct the four velocity fields well.

For the medium noise level, after optimization, the average error of the four fields is reduced by 4.6678%; at this time, the $E_{rms}$ of the velocity fields 2, 3, and 4 for reconstruction before optimization have exceeded 18%, which makes it difficult to accurately restore the original velocity field, but it still has good stability after optimization.

For the high noise level, the average error of the four fields after using optimization is reduced by 8.5388%. At this time, before optimization, the reconstruction effect is greatly affected by the noise, and it is no longer able to correctly reconstruct each velocity field, and its average error of the four fields has already reached 22.4680%, which indicates that its stability is weaker. The optimized region division can still reconstruct the velocity fields correctly.

In summary, the algorithm proposed in this paper not only has high reconstruction accuracy but also has strong stability.

#### 4.2.2. Experiments Based on Simulated Velocity Field

In order to further verify the effectiveness of the optimization algorithm proposed in this paper, this section compares the reconstruction results before and after the optimization of the measured region division based on the simulation model of the velocity field shown in Figure 6.

**Figure 10:**
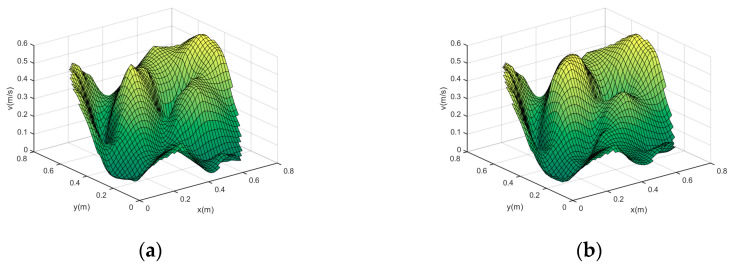
Simulated velocity field reconstruction results before and after optimization. (**a**) Before optimization; (**b**) after optimization.

The simulated flow velocity reconstruction results before and after optimization are shown in Figure 10:

Comparing Figure 10 with Figure 6, we can obtain that the two vortex locations of the original simulation field are (0.53, 0.31) and (0.39, 0.52), the coordinates of the reconstructed vortex before optimization are (0.56, 0.28) and (0.30, 0.45), and the coordinates of the reconstructed vortex after optimization are (0.54, 0.30) and (0.39, 0.46). By comparison, after optimization, the reconstructed velocity distribution is more accurate and better reconstructed.

Table 6 demonstrates $E_{rms}$ of the reconstruction of the simulated velocity field before and after optimization, with reconstructed image pixels 200 × 200.

After comparison, the reconstruction accuracy after optimization is better than that before optimization, and its $E_{rms}$ decreases by 3.8615%, which proves that by optimizing the division of the measured region, the reconstruction can be improved effectively, and the reconstruction accuracy is greatly improved in the velocity field with complex distribution.

In summary, the optimization algorithm of measured region division proposed in this paper can not only effectively improve the reconstruction accuracy of the velocity field in a circular area without changing the original layout of acoustic transducer but also has strong stability.

## 5. Conclusions

In this paper, using AT, a measured region division optimization algorithm for circular area velocity field reconstruction is designed on the basis of the MK-SVD algorithm. Through experimental comparison, the following conclusions can be drawn:

(1) The acoustic path distribution based on multipath effect can realize more accurate velocity field reconstruction. Reconstruction experiments in model and simulated velocity fields show that the reconstruction based on the multipath effect in a circular area utilizes more acoustic transducer paths with a limited number of transducers, which not only reduces the cost of industrial applications, but also enables more comprehensive collection of the velocity information in the area, resulting in better reconstruction results.
(2) Multi-objective-based optimization objectives for measured region division can effectively improve the accuracy reconstruction. In this paper, we optimize the measured region division by designing two optimization objectives, namely the condition number of the coefficient matrix and the uniformity of acoustic path distribution. Experiments show that the algorithm can effectively improve the reconstruction accuracy, and the reconstruction errors in the model velocity field and the simulated velocity field are reduced by 2.0740% and 3.8615%, respectively.
(3) Noise is an important factor affecting the accuracy of velocity field reconstruction. We address this by adding three different levels of Gaussian noise: low, medium, and high. The simulation results show that the optimization algorithm proposed in this paper has stronger stability and can still reconstruct each velocity field correctly under high noise levels. The average errors of the four fields under low, medium, and high noise levels are reduced by 3.3148%, 4.6778%, and 8.5388%, respectively, compared with the pre-optimization errors.
(4) Multi-objective-based optimization objectives for measured region division are also applicable to temperature field reconstruction. Although the optimization algorithm proposed in this paper is based on the reconstruction of the flow velocity field in a circular area, the two sub-optimization objectives in the algorithm as well as the design of the optimization variables do not depend on the flow velocity field, and thus the logic of the optimization algorithm proposed in this paper is equally applicable to the reconstruction of the temperature field in a circular area.

The reconstruction algorithm is the key to the acoustic velocity field reconstruction technique. In addition, the measurement accuracy of TOF will also affect the reconstruction results. For example, in practical engineering applications, if there is a large temperature gradient in the area to be measured, it will lead to an acoustic wave bending effect, which affects the measurement accuracy of TOF. How to measure TOF accurately will be the focus of future research.

## Figures and Tables

**Table 1:** Parameters setting for model velocity fields.

Velocity Field	v (m/s)	$\left(x_{0},y_{0}\right)$ (m, m)	R (m)	σ (m/s)
1	2	(5, 5)	2.5	1
2	2	(3, 4)	2.5	1
3	3, 3	(3, 5) (7, 5)	2.5	1
4	5.5, 7.5	(3, 6) (7, 4)	2.5	1

**Table 2:** Error of reconstruction before and after based on multipath effect in model velocity fields.

Velocity Field	$E_{rms}$	
	Only Direct Paths	Based on Multipath Effect
1	9.5891%	8.9105%
2	15.4611%	14.5554%
3	15.2892%	14.4243%
4	16.1090%	15.4567%
$E_{rms}$ to mean	14.1121%	13.3367%

**Table 3:** Error of reconstruction before and after based on multipath effect in simulated velocity fields.

Velocity Field	$E_{rms}$	
	Only Direct Paths	Based on Multipath Effect
Simulated velocity field	14.0898%	13.4985%

**Table 4:** Error of model velocity fields reconstruction before and after optimization.

Velocity Field	$E_{rms}$	
	Before Optimization	After Optimization
1	8.9105%	7.1190%
2	14.5554%	14.1031%
3	14.4243%	11.9305%
4	15.4567%	11.8980%
$E_{rms}$ to mean	13.3367%	11.2627%

**Table 5:** Error of reconstruction before and after optimization with gaussian noise.

Velocity Field	$E_{rms}$					
	Before Optimization			After Optimization		
	Low	Medium	High	Low	Medium	High
1	10.3264%	11.8939%	18.7679%	7.8429%	8.1858%	9.8350%
2	16.2109%	18.0512%	21.4572%	14.2803%	14.5409%	16.9873%
3	16.2540%	18.3376%	25.0298%	12.2385%	12.8821%	14.1362%
4	17.2301%	18.9342%	24.6171%	12.4002%	12.8970%	14.7851%
$E_{rms}$ to mean	15.0053%	16.8042%	22.4680%	11.6905%	12.1264%	13.9292%

**Table 6:** Error of simulated velocity fields before and after optimization.

Velocity Field	$E_{rms}$	
	Before Optimization	After Optimization
Simulated velocity field	13.4985%	9.6370%

**Figure A1:**
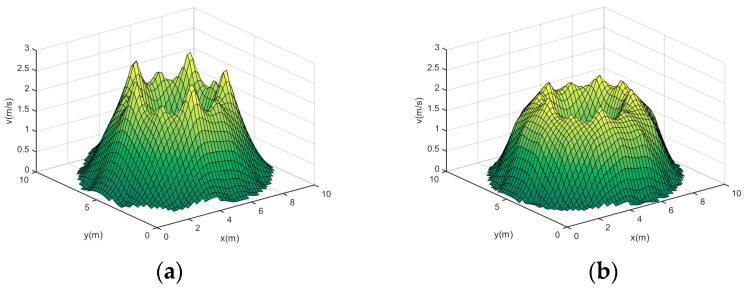

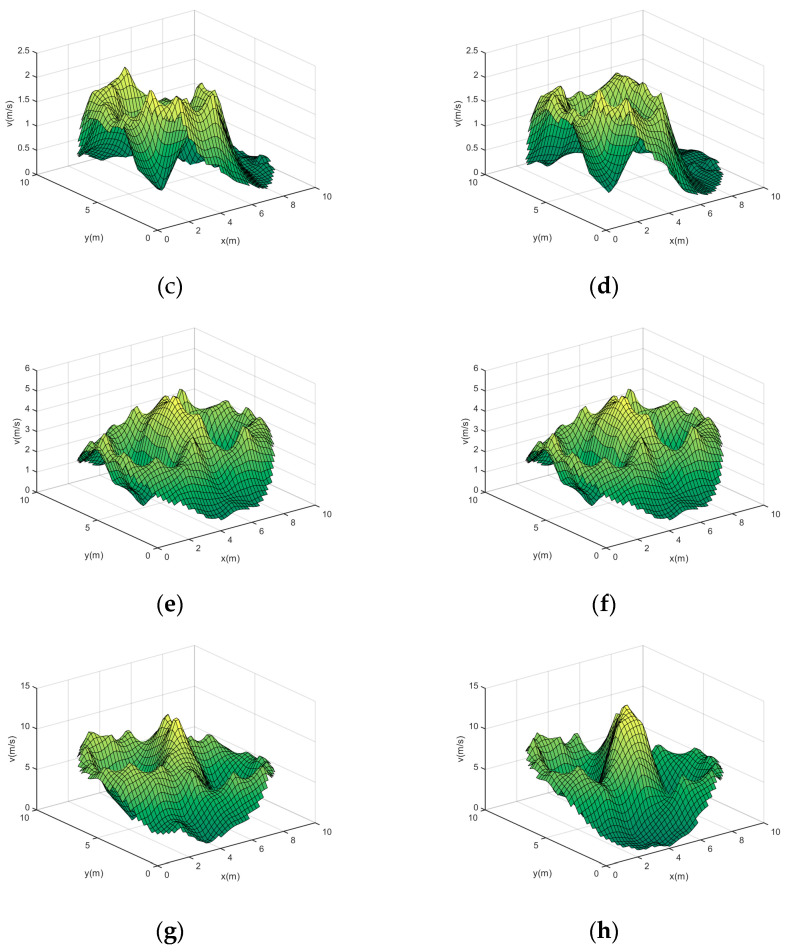
Reconstruction results of model velocity fields under the influence of low level of noise before and after optimization. (**a**) Velocity field 1 before optimization; (**b**) velocity field 1 after optimization; (**c**) velocity field 2 before optimization; (**d**) velocity field 2 after optimization. (**e**) velocity field 3 before optimization; (**f**) velocity field 3 after optimization; (**g**) velocity field 4 before optimization; (**h**) velocity field 4 after optimization.

**Figure A2:**
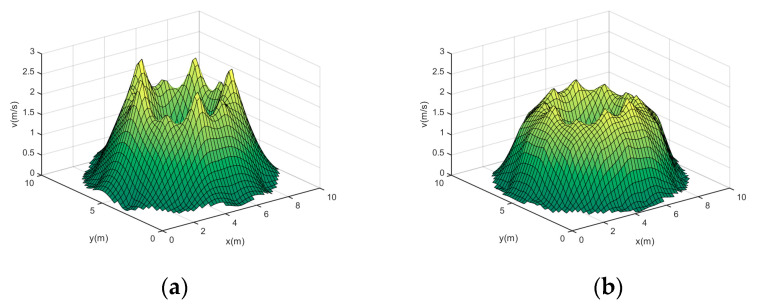

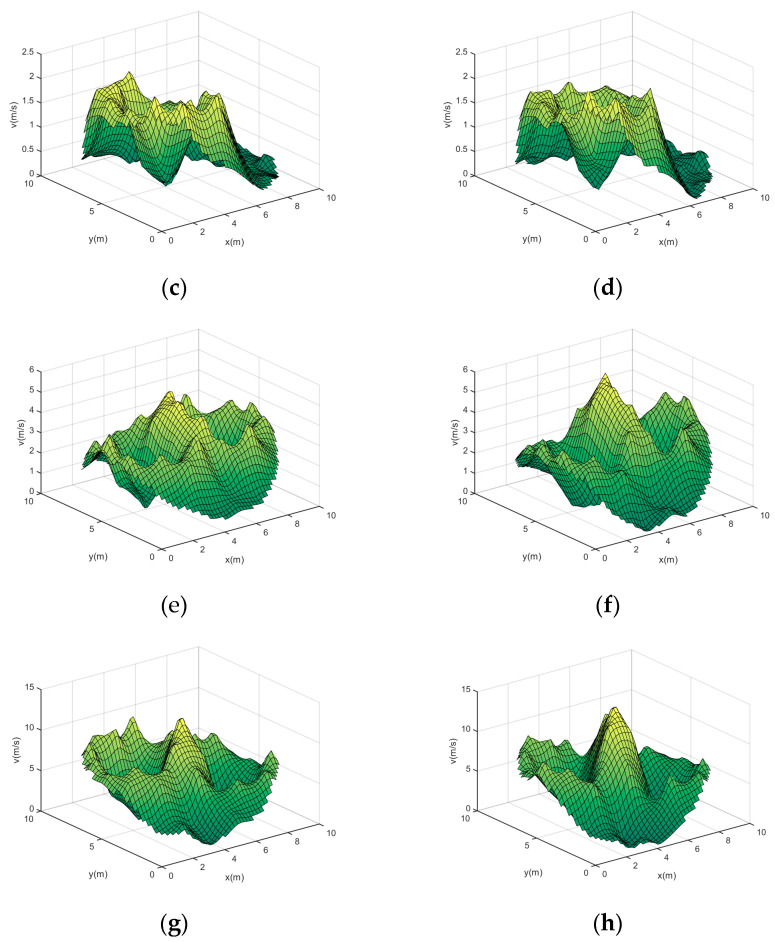
Reconstruction results of model velocity fields under the influence of medium level of noise before and after optimization. (**a**) Velocity field 1 before optimization; (**b**) velocity field 1 after optimization; (**c**) velocity field 2 before optimization; (**d**) velocity field 2 after optimization. (**e**) velocity field 3 before optimization; (**f**) velocity field 3 after optimization; (**g**) velocity field 4 before optimization; (**h**) velocity field 4 after optimization.

**Figure A3:**
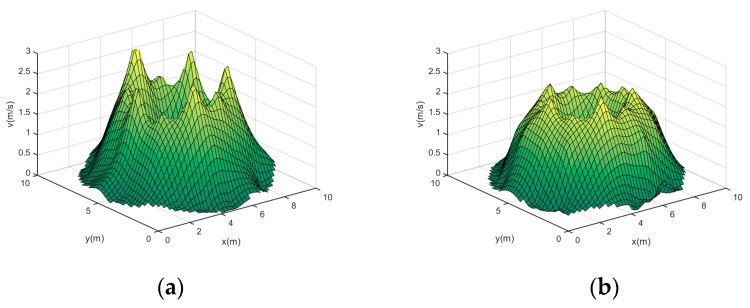

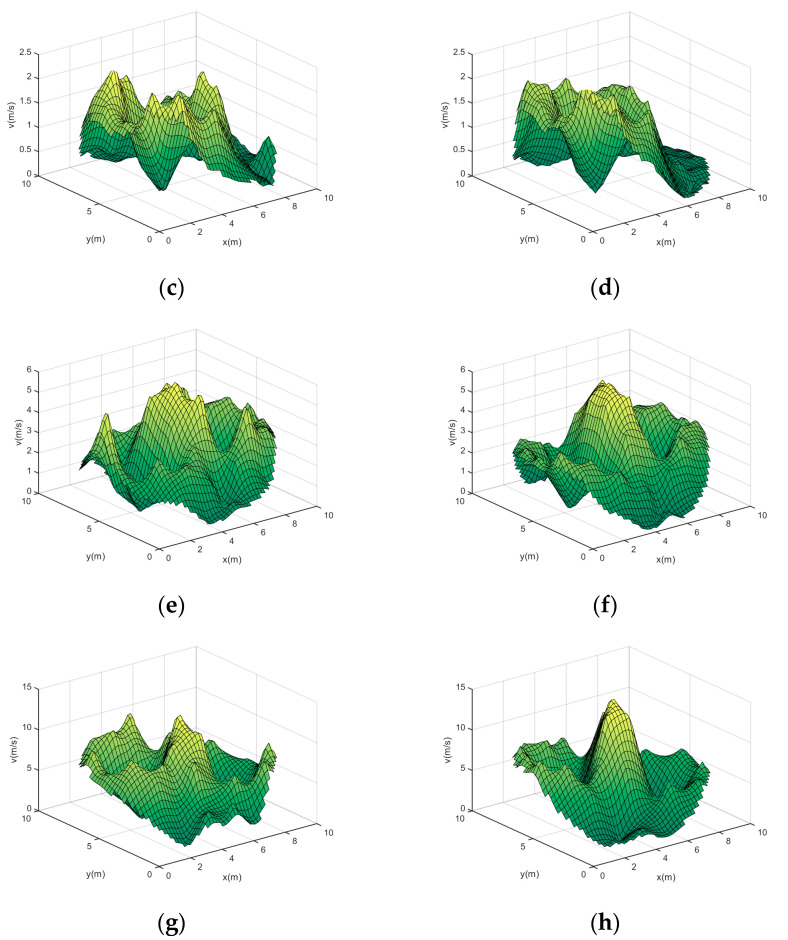
Reconstruction results of model velocity fields under the influence of high level of noise before and after optimization. (**a**) Velocity field 1 before optimization; (**b**) velocity field 1 after optimization; (**c**) velocity field 2 before optimization; (**d**) velocity field 2 after optimization. (**e**) velocity field 3 before optimization; (**f**) velocity field 3 after optimization; (**g**) velocity field 4 before optimization; (**h**) velocity field 4 after optimization.

## Data Availability

Data are contained within the article.

## Appendix A

The flow field reconstruction results before and after optimization under the influence of low noise are shown in Figure A1.

The flow field reconstruction results before and after optimization under the influence of medium noise are shown in Figure A2.

The flow field reconstruction results before and after optimization under the influence of high noise are shown in Figure A3.
